# Optoacoustic imaging of the brain in a cachexia-inducing pancreatic cancer xenograft

**DOI:** 10.3389/fonc.2025.1580640

**Published:** 2025-10-13

**Authors:** Saleem Yousf, Marie-France Penet, Andrew Brannen, Paul Winnard, Yelena Mironchik, Balaji Krishnamachary, Zaver M. Bhujwalla

**Affiliations:** ^1^ Division of Cancer Imaging Research, The Russell H. Morgan Department of Radiology and Radiological Science, The Johns Hopkins University School of Medicine, Baltimore, MD, United States; ^2^ Sidney Kimmel Comprehensive Cancer Center, The Johns Hopkins University School of Medicine, Baltimore, MD, United States; ^3^ iThera Medical, GmbH, Munich, Germany; ^4^ Department of Radiation Oncology and Molecular Radiation Sciences, The Johns Hopkins University School of Medicine, Baltimore, MD, United States

**Keywords:** pancreatic ductal adenocarcinoma, cachexia, optoacoustic imaging, brain, vascular oxygenation

## Abstract

Pancreatic cancer-induced cachexia drives co-morbidities that result in a poor quality of life. To expand understanding of the effects of cachexia on the brain here, for the first time, we used noninvasive oxygen enhanced (OE) multispectral optoacoustic tomography (MSOT) to evaluate the ability of the brain vasculature to respond to oxygen breathing in an established xenograft model of pancreatic cancer-induced cachexia. Studies were performed with mice bearing cachexia inducing Pa04C tumors, non-cachexia inducing Panc1 tumors and non-tumor bearing mice. OE-MSOT identified a reduced oxygen carrying capacity in the brain vasculature of mice with cachexia inducing Pa04C tumors, compared to non-tumor bearing mice, and mice with non-cachexia inducing Panc1 tumors. Brain volumes, quantified in mice with MSOT, were significantly reduced in Pa04C tumor-bearing mice compared to non-tumor bearing mice. Our data have identified the inability of brain vasculature to increase oxygenation in response to oxygen breathing in cachectic mice as a new mechanism that may contribute to cachexia-induced morbidity.

## Introduction

Cachexia is a debilitating syndrome that occurs in chronic heart failure, chronic obstructive pulmonary disease, rheumatoid arthritis, and in certain types of cancer (1, 2). In the international consensus statement of 2011, cancer cachexia was defined as ‘a multifactorial syndrome characterized by an ongoing loss of skeletal muscle mass with or without loss of fat mass that cannot be fully reversed by conventional nutritional support and leads to progressive functional impairment’ (3). The onset of cachexia has been described as unintentional weight loss exceeding 5% in the previous 6 months, or a body-mass-index (BMI) <20 kg/m^2^ with ongoing weight loss of >2%, or sarcopenia and ongoing weight loss of >2% (3). Cancer cachexia is observed most frequently in advanced pancreatic ductal adenocarcinoma (PDAC) and lung cancer, although it is also observed in liver, ovarian, colorectal and head and neck cancers (4–9). In PDAC patients, cachexia has been associated with reduced physical function, lower response rates to chemotherapy and radiotherapy, and lower survival rates (4, 10), with approximately one-third of PDAC patients dying from cachexia related complications (10). In addition, PDAC patients with cachexia demonstrate poor treatment tolerance and decreased survival after pancreatectomy (4, 11).

The high prevalence of cachexia in PDAC is attributed to tumor-released factors, the disruption of pancreatic function, and the close interaction between the pancreas and the gut (12). Altered pancreatic function was found to drive early cachexia development in pancreatic cancer preclinical models (13). PDAC patients characteristically have elevated levels of pro-inflammatory cytokines, such as IL-6 and TNF-alpha that drive systemic inflammation, which is one of the defining conditions of cachexia (14, 15). Previous studies have detected overexpression of pro-inflammatory cytokines including TNF-α and IL-1β in the hypothalamus of cachexia models (16, 17) that likely contributes to the modulation of appetite.

In a study with patient derived PDAC cell lines, we found, relative to non-tumor bearing (NTB) normal mice and non-cachexia inducing Panc1 tumors, that Pa04C tumors induced significant body weight loss (18). In addition, Pa04C tumor-bearing mice, but not Panc1 tumor-bearing mice, activated a MuRF1 (Muscle RING-finger protein-1) promoter reporter in myoblasts grafted onto the biceps femoris muscle (18). MuRF1 is a muscle specific E3 ubiquitin ligase that is upregulated during muscle atrophy (19, 20). Brain weights were significantly reduced in Pa04C mice compared to NTB normal mice or mice bearing non-cachexia inducing Panc1 tumors (21). A cachectic brain metabolic signature in these mice was characterized by depleted choline and increased glutamine and formate (21). Altered glutamine metabolism and brain weight loss in these cachectic mice led us, in the present study, to evaluate brain oxygenation, hemodynamics and volume using noninvasive optoacoustic imaging. Hypoxia can promote the uptake of glutamine by increasing the levels of glutamine transporters (22, 23). Anemia that is frequently observed in PDAC can also contribute to alterations in vascular hemodynamics (24, 25). While the precise mechanisms underlying anemia in PDAC remain unclear, contributing factors such as iron deficiency have been documented in over 60% of PDAC patients, identifying its role as a major contributor to anemia in this cancer type (25).

Multispectral optoacoustic tomography (MSOT) or optoacoustic tomography (OT) imaging is an emerging non-invasive hybrid modality that combines the high contrast and spectral specificity of optical imaging with the high spatial resolution and penetration depth of ultrasound (US) imaging, providing structural and functional images of tissues in real-time (26). MSOT provides a distinct advantage for measuring deep tissue oxygenation in mouse models. Unlike conventional optical techniques that are limited by shallow penetration, MSOT utilizes near-infrared light to generate optoacoustic signals capable of penetrating ~ 4–5 cm into tissue, allowing for high-resolution, non-invasive oxygenation measurements at these depths that are clearly sufficient for the mouse brain. The use of spectral unmixing allows the differentiation of oxyhemoglobin (HbO_2_) from deoxyhemoglobin (Hb), enabling precise and accurate quantification of oxygen saturation, even in complex and heterogeneous tissue environments. Numerous studies have successfully utilized MSOT to assess tissue oxygenation at these depths in both preclinical and clinical settings (27–37). During MSOT imaging, tissues are illuminated with pulsed laser light at multiple wavelengths, typically in the near-infrared range, where HbO_2_ and Hb exhibit different absorption characteristics. The absorbed light induces thermoelastic expansion, generating ultrasonic waves that are detected by ultrasound transducers. These signals are then processed using spectral unmixing algorithms that separate HbO_2_ and Hb based on their unique spectral signatures, allowing for the calculation of their relative concentrations. The sO_2_ is determined by calculating the ratio of HbO_2_ to total hemoglobin, using the formula: sO_2_ = [HbO_2_]/[HbO_2_+Hb]. By switching the respiratory gas from medical air (21% oxygen) to 100% oxygen at 760 mm Hg, static measurements of total hemoglobin, and oxy- and deoxyhemoglobin can be combined with dynamic oxygen-enhanced optoacoustic tomography (OE-MSOT), to detect changes in hemoglobin oxygenation. Dynamic OE-MSOT measurements have been demonstrated to correlate with histopathologic analyses of tumor vascular function (38, 39).

We performed OE-MSOT to monitor changes in hemoglobin oxygenation in the brains of cachectic Pa04C tumor-bearing mice, compared to NTB normal mice, and mice with non-cachexia-inducing Panc1 tumors. We additionally performed CD31 immunostaining of brain sections that identified changes in brain vasculature with cachexia. Our data highlight the significant physiological and structural changes that occur in the brain in mice with cachexia-inducing tumors, identifying a reduction in brain vascular oxygenation and volume as potential factors contributing to morbidity. These insights may lead to approaches such as increasing brain oxygenation to improve quality of life and reduce morbidity. While MSOT imaging is limited by depth penetration, translational imaging techniques such as perfusion MRI may be applied to detect changes in brain hemodynamics and brain volume for cachexia diagnosis and for detecting therapeutic response to interventions in translational applications.

## Materials and methods

### Tumor xenografts

The Panc1 cell line, derived from a 56-year-old male patient with PDAC, was acquired from American Type Culture Collection (ATCC, Rockville, MD). The Pa04C cell line, obtained from a 59-year-old male patient with PDAC lung metastasis, was generously provided by Dr. Anirban Maitra. Both cell lines were cultured in Dulbecco’s Modified Eagle Medium (DMEM) supplemented with 10% fetal bovine serum under standard incubation conditions of 37 °C, 5% CO_2_, and a humidified environment.

All animal studies were performed in strict compliance with protocols approved by the institutional Animal Care and Use Committee. These practices adhered to the guidelines outlined in the “Guide for the Care and Use of Laboratory Animals” published by the National Institutes of Health.

Tumor xenografts were derived from the inoculation of 2 × 10^6^ cells suspended in 50 μL of Hanks solution in the right flank of six to eight-week-old male immunodeficient nude mice. MSOT hemodynamic and brain volume studies were performed with 5 NTB normal mice, 5 Panc1 tumor-bearing mice, and 5 Pa04C tumor-bearing mice.

Mice were weighed every 2–3 days at which time tumor volumes, calculated as 0.524 x length x width x depth, were recorded using a digital caliper. The brain imaging studies were performed once tumor volumes were approximately 450–500 mm³ at approximately 6 to 9 weeks from inoculation.

### Multispectral optoacoustic tomography

MSOT imaging was performed using an MSOT inVision 512-echo scanner (iThera Medical, GmbH, Munich, Germany.) that integrates simultaneous tomographic optoacoustic and tomographic ultrasound acquisitions. This small animal imaging scanner is equipped with a 512- toroidally-focused ultrasound (US) transducer array operating at a central frequency of 5 MHz and spanning a circular arc of 270° to effectively detect optoacoustic signals. Both optoacoustic and ultrasound images are co-registered as they are acquired using the same tomographic ultrasound transducer array. Briefly, light excitation was provided with a tunable optical parametric oscillator (OPO) pumped by an Nd: YAG laser. Excitation pulses with a duration of 9 ns at wavelengths ranging from 700 nm to 900 nm at a repetition rate of 10 Hz, wavelength tuning speed of 10 ms, and a peak pulse energy of 100 mJ at 720 nm were used. The ten arms of a fiber bundle were positioned evenly around the animal to create a ring-shaped illumination around the mouse body coinciding with the US detection plane.

Mice were anesthetized with 2% isoflurane using medical air. For MSOT brain imaging, the animals were placed in a supine position in a customized animal holder (iThera Medical, GmbH, Munich, Germany.), wrapped in a thin polyethylene membrane. A thin layer of US gel was applied to the skin for better acoustic coupling to the membrane prior to imaging. The holder was then placed within the water chamber of the MSOT scanner maintained at 34 °C, and the mouse was allowed to acclimatize for 15 min. Although the MSOT scanner used in our study does not have an integrated respiration or ECG monitoring unit, respiration was assessed visually using the scanner’s built-in camera to observe thoracic and abdominal movements of the animal while under anesthesia. Based on these movements, the respiratory rate of mice was approximately maintained in the range of 70–80 breaths per minute by manually adjusting the anesthesia. During preparation and acclimatization, mice were breathing medical air (21% O_2_) at 160 mmHg. The mouse was moved through the transducer array along its axis and cross-sectional image slices of the region of interest were acquired, with a step size of 0.5 mm. Continuous imaging commenced using 6 wavelengths (700, 730, 760, 800, 860 and 900 nm) with an average of 10 pulses per wavelength. During OE-MSOT imaging, a single slice at the center of the brain was continuously imaged throughout the dynamic gas challenge. The same anatomical location was used in all the mice. Each mouse was continuously imaged, first during 3 minutes of breathing regular medical air, followed by breathing 100% oxygen for another 8–10 minutes (3 min at 21% O_2_; 8–10 min at 100% O_2_).

### MSOT image processing

Data analysis of the MSOT images was performed using viewMSOT (Version 4.0.2.0, iThera Medical, GmbH, Munich, Germany), which enabled the complete imaging workflow from data acquisition to image reconstruction, spectral unmixing, visualization and quantification. MSOT images were reconstructed from the raw data using a back-projection algorithm at a resolution of 75 μm. Once the multispectral data were acquired, signals corresponding to different chromophores were separated using spectral unmixing algorithms. Spectral unmixing was performed using a linear regression method within the ViewMSOT software. This process enables the separation of Hb and HbO_2_ based on their unique absorption spectra in the near-infrared range. The algorithm assigns the appropriate reference spectrum to each pixel in the image, allowing for the precise quantification of oxygen saturation (sO_2_) by calculating the ratio of HbO_2_ to total hemoglobin (HbT). A region of interest (ROI) was manually drawn over the entire cross-sectional area of the brain, excluding regions in the field of view that contained large blood volume such as the superior sagittal sinus and temporal arteries, on concurrently acquired optoacoustic images of the brain to determine the spectral signal. Within each ROI, the mean of the highest 10% values was used to analyze hemodynamic parameters. Analyzing the top 10% of the signal, instead of the overall mean, reduced the influence of outliers at the high and low ends of the dynamic range that could bias the mean data and overall trend. In this approach, quantification was performed for pixels with values within the top 10 % of the dynamic range. The limitations of this quantification approach were carefully considered by excluding regions in the field of view that contained large blood volume such as the superior sagittal sinus and temporal arteries, to restrict the dynamic range to the brain parenchyma. This approach provides a window for which the response of the oxygen challenge can be graphically appreciated without bias from outliers and low-responding/non-responding pixels. The average oxygen saturation, calculated for each pixel within the ROI during both air and oxygen breathing periods, was represented as sO_2_^MSOT^(Air) and sO_2_^MSOT^(O_2_), respectively. The amplitude of response to the oxygen gas challenge, ΔsO2^MSOT^, was calculated for each pixel by subtracting sO_2_^MSOT^(Air) from sO_2_^MSOT^(O_2_). Identical reconstruction parameters, such as field of view, speed of sound, pixel size, high/low pass filters, and spectral unmixing parameters were consistently applied to all the data sets.

To calculate the brain volume, cross-sectional 2D images were acquired at a step size of 0.5 mm along the z-axis to cover the whole brain. Each 2D slice was reconstructed using the back projection algorithm. The reconstructed 2D slices were then processed in viewMSOT software to create a 3D volume by stacking the 2D slices along the z-axis. ROIs were manually drawn along brain boundaries on each 2D slice, using ultrasound images for anatomical accuracy. Ultrasound imaging provided anatomical details essential for identifying the brain’s structural boundaries. Interpolation between slices was applied wherever necessary to ensure a continuous 3D structure. Orthogonal planes in the x-y, x-z, and y-z orientations were used to define the 3D ROI to encompass the entire brain (**Supplementary Figure S1**). Once the ROI was defined across the 3D volume, the software calculated the brain volume within the defined region.

Unlike MRI that offers superior spatial resolution, the spatial resolution of ultrasound/optoacoustic imaging is lower that makes it challenging to delineate small anatomical boundaries such as the olfactory bulb and cerebellum. However, to ensure accuracy and reproducibility in our volumetric measurements, we used standardized animal positioning, maintained identical acquisition parameters, and confirmed slice positions across animals through anatomical landmark referencing using both the co-registered ultrasound and photoacoustic imaging data from the MSOT scanner. While the volumetric precision is lower than for MRI, volume changes between groups can be identified under these standardized imaging conditions. All coronal brain slices for the representative mouse brain are presented in **Supplementary Figure S2**.

### OE-MSOT data analysis

For the quantification of ΔsO_2_^MSOT^, OE-MSOT data were subjected to non-linear regression analysis using GraphPad Prism (version 6.0, GraphPad Software). This curve-fitting approach rather than a manual approach was selected to avoid any operator bias. As shown in **Supplementary Figure S3**, data were fitted to a “plateau followed by a one-phase association” model within GraphPad Prism software. This built-in model provided a best fit for the sO_2_ response curve, allowing for the extraction of key parameters that describe the kinetics of oxygen saturation changes in the tissue.

The equation used for the curve-fitting was defined as follows:


Y= IF (X<X0, Y0,Y0 + (Plateau−Y0)*(1 − exp(−K*(X−X0))))

*where:*

*Y* is the MSOT signal level, *X* is time, *X0* represents the time at which the respiratory gas was switched from air to oxygen, *Y0* is the average Y value up to time *X0*, *Plateau* is the Y value at infinite times, expressed in the same units as Y, and *K* is the rate constant.

The amplitude of response, ΔsO_2_^MSOT^, was determined using the *Span* parameter calculated by GraphPad Prism software, defined as the difference between *Plateau* and *Y0*. This parameter quantifies the magnitude of change in oxygen saturation following the oxygen challenge, providing a measure of the dynamic response of brain vasculature to altered oxygen conditions.

### CD31 immunostaining

Formalin-fixed, paraffin-embedded sections of brains from NTB (n=3), Pa04C (n=3) and Panc1 (n=3) tumor-bearing mice were deparaffinized and processed using a standard protocol (40) Following standard endogenous peroxidase blocking and non-specific protein blocking, tissue sections were incubated with the rat monoclonal CD31 antibody (Dianova, Hamburg, Germany, 1:30 dilution) overnight at 4°C. Following this, sections were processed with Elite rat specific ABC Vector staining kit (Vector Laboratories, Newark, CA, Cat. No.- PK-6104). Slides were stained with 3,3′-diaminobenzidine (DAB) and counterstained with hematoxylin. High-resolution digital scans of the stained brain sections were obtained using ScanScope (Aperio, Vista, CA). Quantification was performed using the ImageScope software Positive Pixel Count V9 algorithm supplied by the manufacturer. The area occupied by CD31 positive pixels was normalized to the area of brain tissue to obtain the percent CD31 fraction in each section.

### Statistical analysis

To assess the normality of our datasets, we conducted the Kolmogorov-Smirnov (K-S) test for ΔsO_2_^MSOT^ (Control: *p* = 0.88, Panc1: *p* = 0.23, Pa04C: *p* = 0.78), weight change (Control: *p* = 0.07, Panc1: *p* = 0.50, Pa04C: *p* = 0.25), and volume (Control: *p* = 0.29, Panc1: *p* = 0.18, Pa04C: *p* = 0.99). All datasets yielded p-values greater than 0.05, indicating no significant deviation from a normal distribution. Given that the normality assumption was met, a Student’s two-sided t-test was conducted to identify statistically significant differences between groups. For multiple comparisons, one-way analysis of variance (ANOVA) followed by *post hoc* tests (Holm–Sidak method or Fisher’s least significant difference (LSD) test) was performed to determine statistically significant differences among groups, with p < 0.05 considered significant.

## Results

### Body weight evaluation

**Figure 1:**
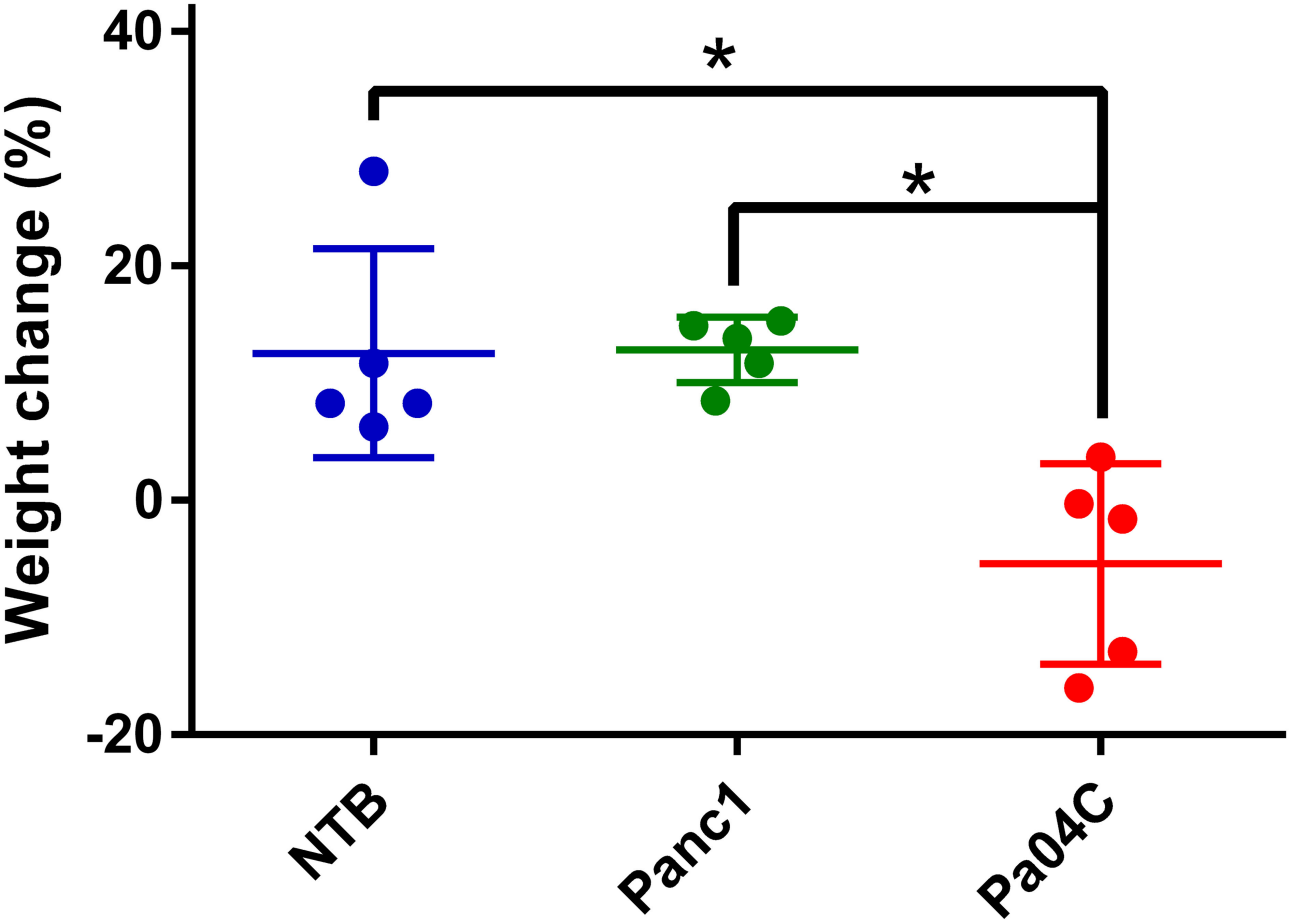
Body weight changes. Body weight change of each mouse relative to initial weights as percent gains or losses once tumor volumes were approximately 450–500 mm³. Final weights were measured at the time of imaging. Statistical analyses were performed using one-way ANOVA followed by Holm-Sidak’s multiple comparisons test. *denotes statistical significance with p < 0.05. Values represent Mean +/- SD.

Cachexia-inducing Pa04C tumors induced significant weight loss, in contrast to the weight gains seen in non-cachectic Panc1 mice and NTB mice as shown in **Figure 1** for weights measured at the end of the study. All groups were provided with the same standard nutrition throughout the experiment suggesting that the weight loss was involuntary.

### Cachexia and changes in brain volume

**Figure 2:**
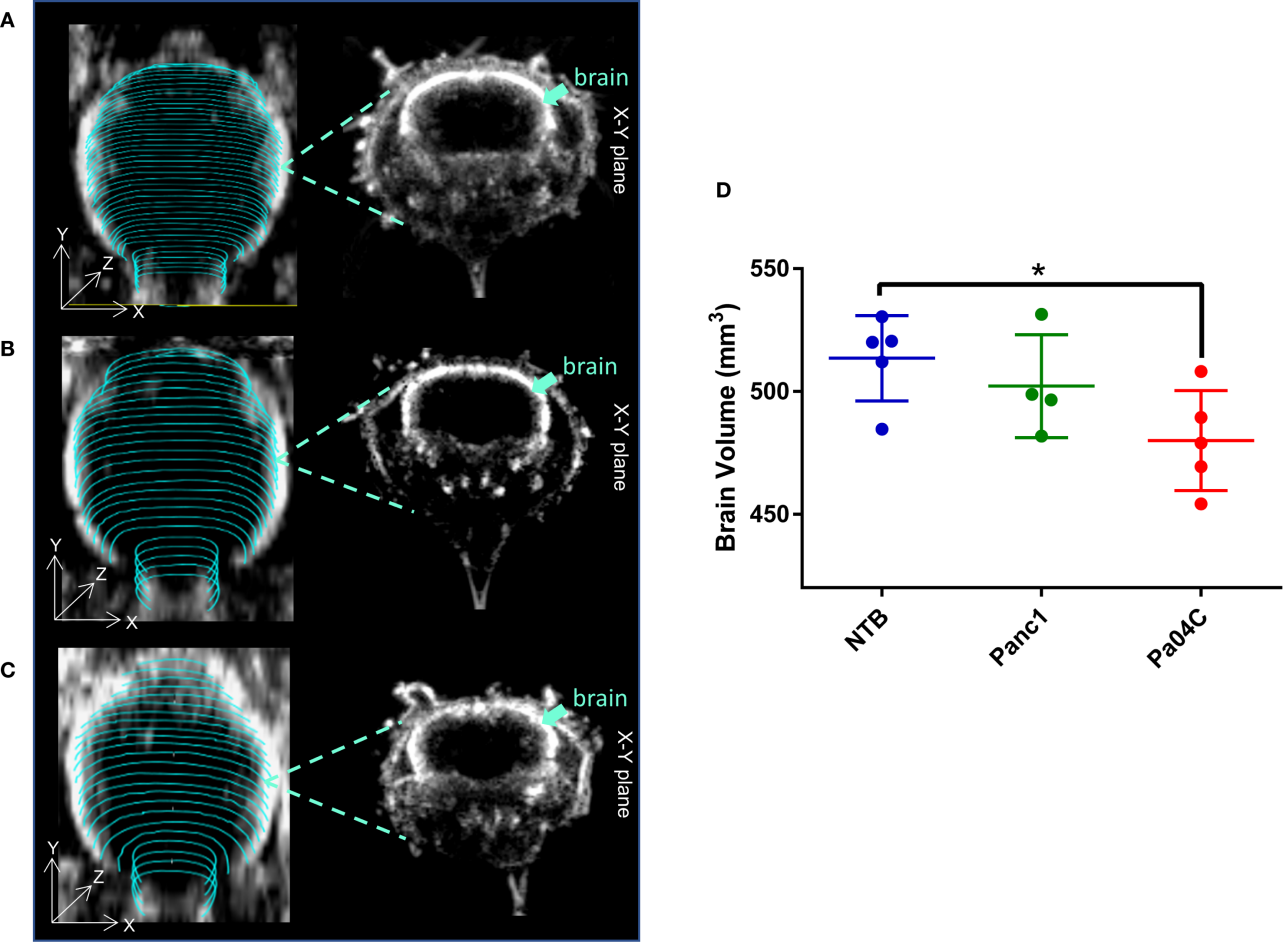
Impact of cachexia on brain volume. Representative 3D brain ultrasound (US) images of **(A)** a non-tumor-bearing (NTB) normal mouse, **(B)** a Panc1 tumor-bearing mouse, and **(C)** a Pa04C tumor-bearing mouse. Regions of interest (ROIs) outlined in cyan were delineated on transaxial cross-sectional brain slices using B-mode ultrasound images for brain volume quantification. Corresponding midbrain transaxial cross-sectional ultrasound images are shown adjacent to the 3D US images. **(D)** Summary of brain volume for NTB normal mice and for Panc1 and Pa04C tumor-bearing mice. The reduction in brain size is evident in the Pa04C tumor-bearing mouse compared to NTB and Panc1 tumor-bearing mice. Brain volumes were determined at the time of imaging when tumor volumes were approximately 450–500 mm³. Statistical analyses were performed using one-way ANOVA followed by Holm-Sidak’s multiple comparisons test. *denotes statistical significance with p < 0.05. Values represent Mean +/- SD.

Pa04C tumors induced a significant decrease of brain volume compared to NTB normal mice as shown in the representative ultrasound images in **Figures 2A-C**, and summarized in **Figure 2D**. On average, the mean brain volumes of NTB normal and Panc1 tumor-bearing non-cachectic mice were approximately 513 mm^3^ and 502 mm^3^, respectively. In comparison, the mean brain volume of Pa04C mice was approximately 479 mm^3^ at the time of imaging.

### *In vivo* MSOT hemodynamic changes during respiratory gas challenge

**Figure 3:**
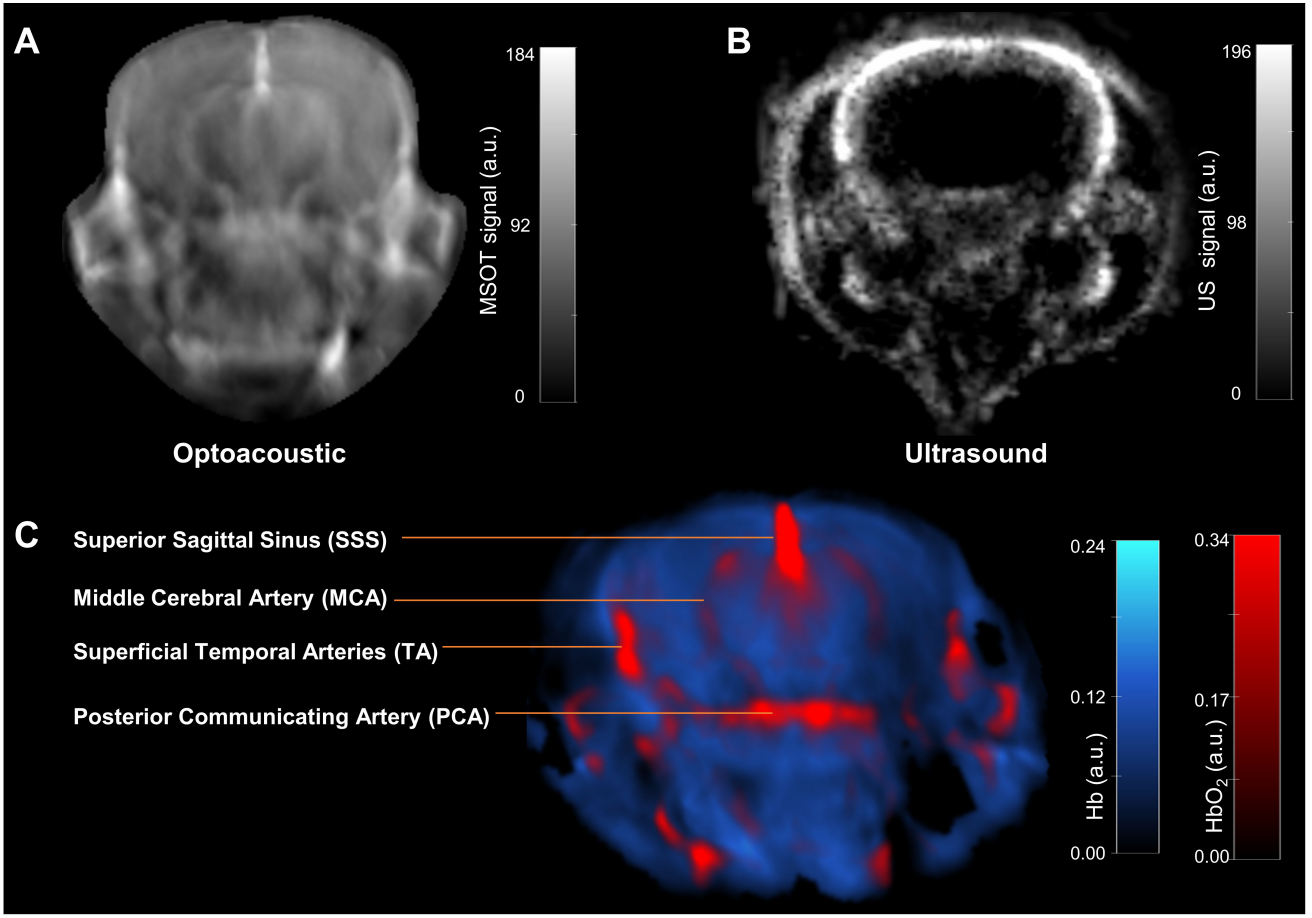
*In vivo* MSOT anatomical and functional imaging of a live intact mouse brain. **(A)** Transaxial cross-sectional background (single wavelength: 850 nm) anatomical photoacoustic image of an intact brain from a NTB normal living mouse. **(B)** Corresponding transaxial cross-sectional ultrasound brain image. **(C)** The multispectral data unmixed for the oxygenated (HbO_2_) and deoxygenated (Hb) hemoglobin are overlaid on the single wavelength (850 nm) image in red and blue, respectively. Brain structures such as the superior sagittal sinus (SSS), the middle cerebral artery (MCA), superficial temporal arteries (TA) and the posterior communicating artery (PCA) are visible.

**Figure 4:**
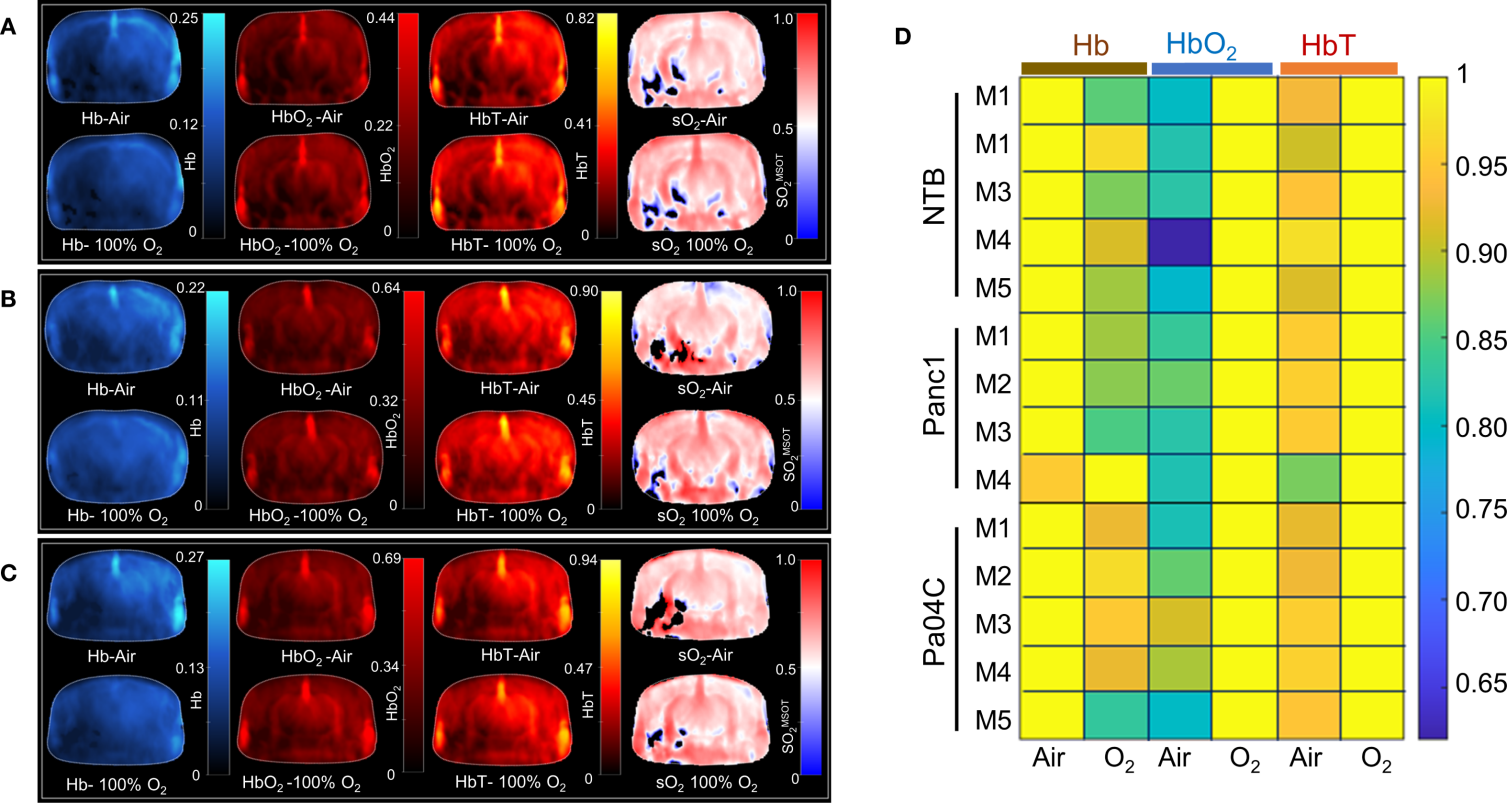
Hemodynamic parameters and heat map. Representative brain images depicting hemodynamic parameters, including Hb, HbO_2_, total Hb, and sO_2_^MSOT^, under air and oxygen breathing conditions in **(A)** NTB, **(B)** Panc1 tumor-bearing mice, and **(C)** Pa04C tumor-bearing mice. **(D)** Heat map of Hb, HbO_2_, and total Hb under air and oxygen respiratory gas, with values normalized to the highest value in each dataset for each parameter and set to 1. The black areas in the sO2 maps in **(A-C)** represent regions where the signal was insufficient for the algorithm to compute the sO_2_.

A representative trans-axial cross-sectional single wavelength MSOT image, acquired at 850 nm through intact skin and skull, of the brain from a living NTB normal mouse presented in **Figure 3A** reveals the major blood vessels and other anatomical features in the brain. The corresponding trans-axial US brain image is presented in **Figure 3B**. Spectral unmixing of signals was applied to identify the presence of Hb and HbO_2_, as shown in the representative image in **Figure 3C**. The absorption spectra of Hb and HbO_2_ utilized for spectral unmixing and calculating sO_2_ are shown in **Supplementary Figure S4**. Prominent photoacoustic signals were clearly detected in major blood vessels deep within the brain, such as the superior sagittal sinus (SSS), the middle cerebral artery (MCA), the superficial temporal arteries (TA) and the posterior communicating artery (PCA) through the intact skull and skin (**Figure 3C**). To quantify brain oxygenation and hemodynamics, an ROI encompassing the brain but excluding major blood vessels was defined, as shown in **Supplementary Figure S5**. **Figures 4A-C** display representative images of single wavelength cross-sectional images of the hemodynamic parameters Hb, HbO_2_, HbT and sO_2_ from the brain of NTB normal mice (**Figure 4A**), Panc1 mice (**Figure 4B**) and Pa04C mice (**Figure 4C**) during the air-oxygen gas challenge. The heatmap displayed in **Figure 4D**, illustrates the pattern of alterations in hemodynamic parameters across the three groups, providing an overview of the hemodynamic changes in NTB normal mice, Panc1 mice, and Pa04C mice before and after the respiratory air/oxygen gas challenge, with data normalized to the highest value within the dataset for each parameter. The values for Hb under air breathing conditions, and for HbT and HbO_2_ under oxygen breathing, were set to 1, creating a relative scale for analyzing all hemodynamic parameters between the two breathing conditions. The heatmap identified a reduction in deoxygenated hemoglobin levels during the air/oxygen gas challenge in Pa04C mice when compared to NTB normal mice and Panc1 mice. In contrast, an elevation in hemoglobin levels was observed during the air/oxygen gas challenge in NTB normal mice and Panc1 mice compared to Pa04C mice.

### OE-MSOT identifies differences in brain vascular function

**Figure 5:**
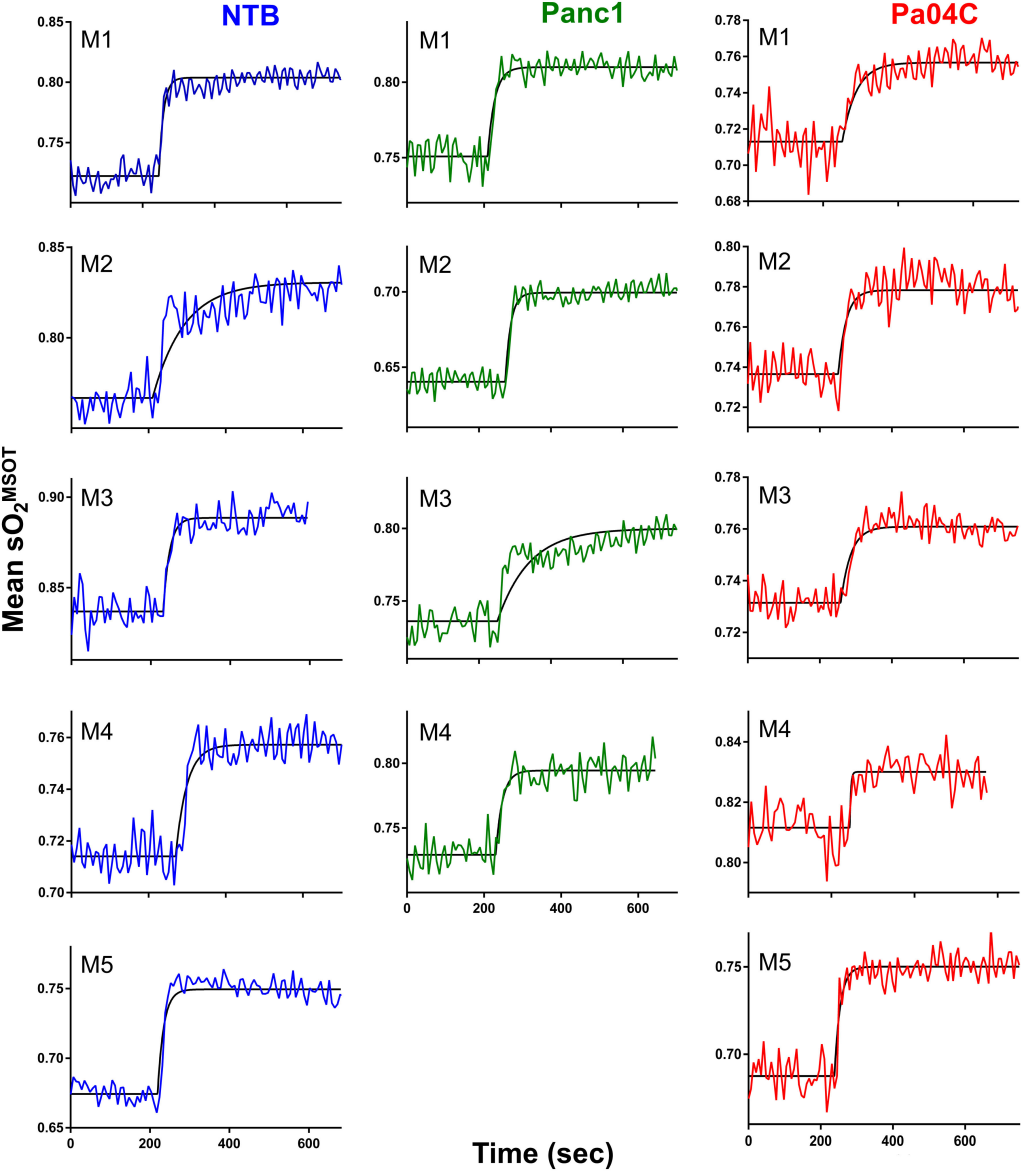
Oxygen-enhanced optoacoustic tomography (OE-MSOT) signal. Kinetic curves displaying the mean sO_2_^MSOT^ values obtained from the intact brains of living mice in NTB (n=5), Pa04C (n=5), and Panc1 (n=4) groups during a 100% oxygen gas challenge. The OE-MSOT signals for NTB mice are depicted by blue curves, Panc1 tumor-bearing mice by green curves, and Pa04C tumor-bearing cachectic mice by red curves. A substantial reduction in ΔSO2^MSOT was observed in the OE-MSOT kinetic curves of the brains in cachectic Pa04C tumor-bearing mice compared to both NTB and Panc1 tumor-bearing mice.

**Figure 6:**
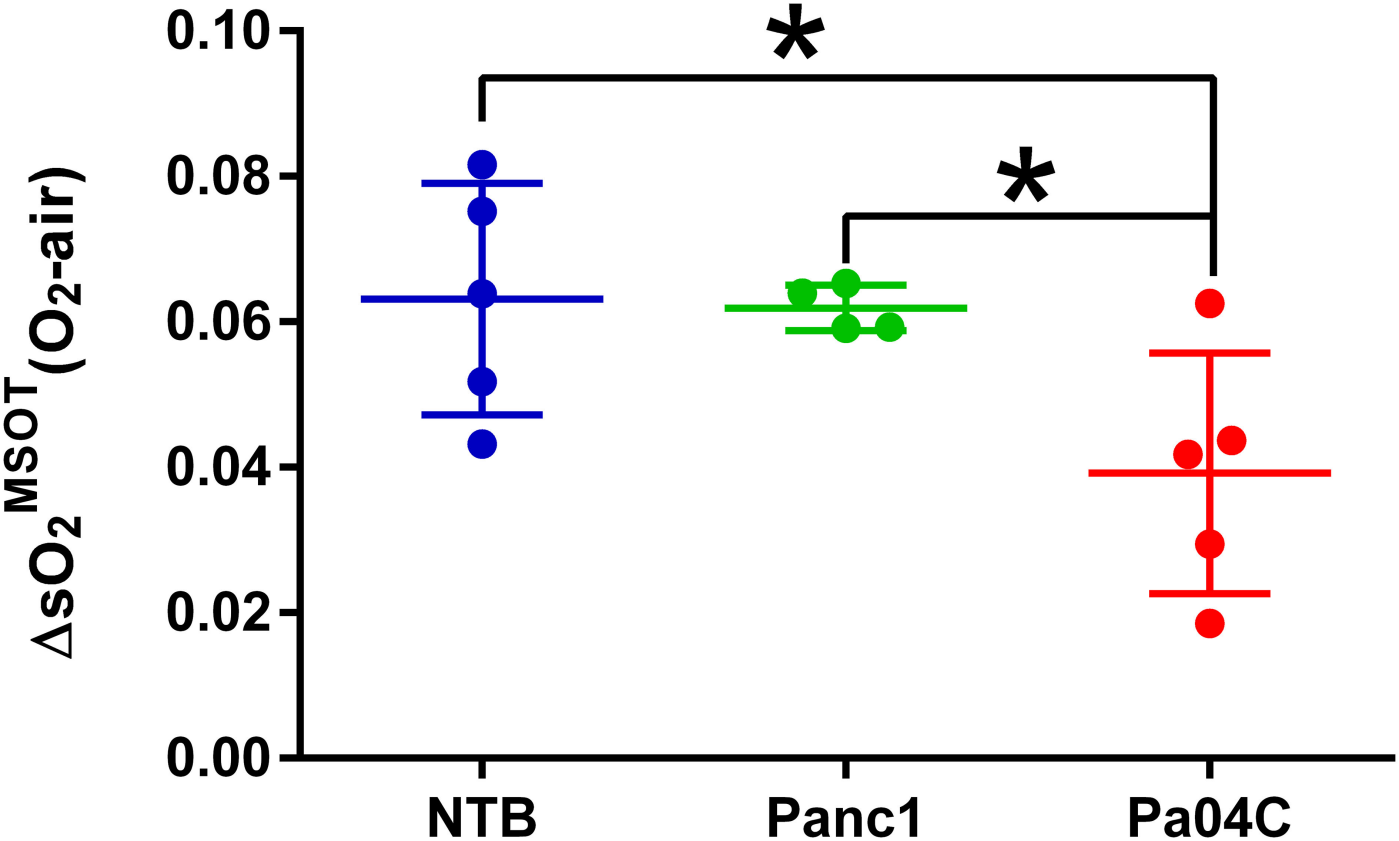
Quantitation of ΔO_2_^MSOT^. Summary of brain ΔO_2_^MSOT^ for NTB and Panc1 and Pa04C tumor-bearing mice. Each data point represents the individual ΔO_2_^MSOT^ value for each mouse within each group. The reduction in ΔO_2_^MSOT^ is evident in Pa04C tumor-bearing mice compared to NTB and Panc1 tumor-bearing mice. Statistical analyses were performed using one-way ANOVA followed by Holm-Sidak’s multiple comparisons test. *denotes statistical significance with p < 0.05. Values represent Mean +/- SD.

Significant differences in response to the O_2_ breathing challenge were clearly identified in multispectral optoacoustic tomography (MSOT)-derived oxygen saturation (sO_2_^MSOT^) in Pa04C tumor-bearing cachectic mice compared to Panc1 tumor-bearing non-cachectic mice and NTB normal mice (**Figure 5**). During the respiratory gas challenge, we observed higher oxygen saturation in NTB normal and Panc1 tumor-bearing mice compared to cachectic Pa04C-bearing mice. OE-MSOT kinetic curves in **Figure 5** were used to calculate the amplitude of response to the oxygen gas challenge, ΔsO_2_^MSOT^. ΔsO_2_^MSOT^ was significantly lower in Pa04C cachectic mice compared to NTB normal and Panc1 mice (**Figure 6**).

### CD31 immunostaining identifies changes in brain vasculature

**Figure 7:**
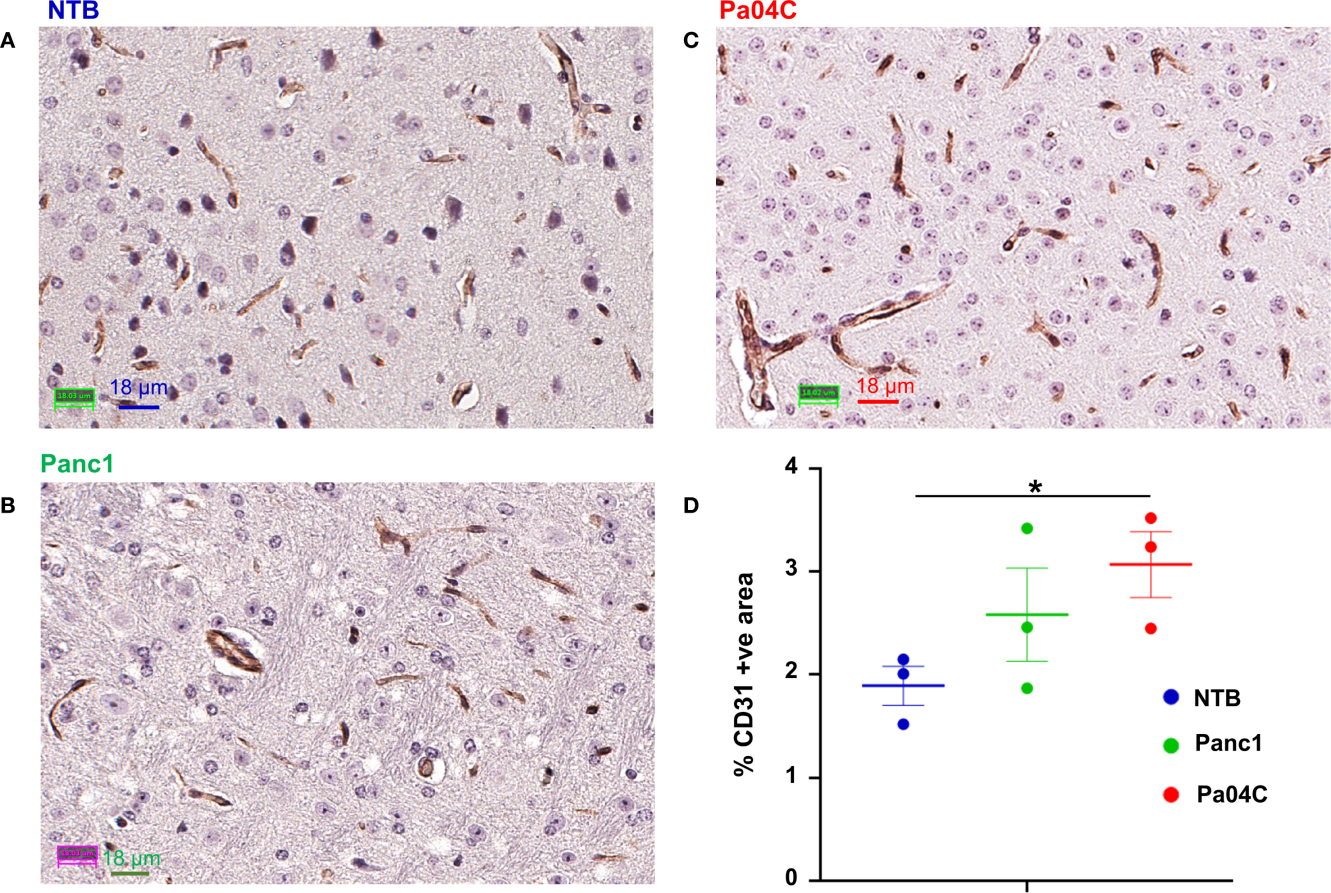
CD31 staining of brain sections. Representative 40X images of brain sections from **(A)** NTB mice, **(B)** Panc1 tumor-bearing mice, and **(C)** Pa04C tumor-bearing mice stained for the endothelial marker CD31. **(D)** Quantification of area occupied by CD31 positive pixels normalized to the total area in NTB (n= 3, blue), Panc1 (n=3, green) and Pa04C (n=3, red) mice. Statistical analyses were performed using one-way ANOVA followed by Fisher’s LSD multiple comparisons test. *denotes statistical significance with p < 0.05. Values represent Mean +/- SEM.

Immunostaining of NTB normal brain, Panc1 and Pa04C tumor-bearing mouse brain for the endothelial marker CD31 showed significant differences in the vasculature. These results summarized in **Figure 7** identified an increase of percent CD31 in the brain of Pa04C tumor-bearing mice compared to NTB normal mice (3.07 ± 0.30 vs 1.89 ± 0.18, p=0.03). There was no significant difference in CD31 expression in the brains of mice with Panc1 and Pa04C tumors or between NTB normal and Panc1 tumor-bearing mice.

## Discussion

We identified a significant decrease of brain volume and a significant reduction of the oxygen carrying capacity within brain vasculature with noninvasive MSOT imaging in our cachexia-inducing PDAC xenograft model. To the best of our knowledge, these are the first studies reporting changes in brain volume and brain vascular oxygenation with cachexia. These studies open new avenues for understanding the physiological underpinnings of cachexia and its impact on brain oxygenation dynamics.

Consistent with the previously established ability of Pa04C tumors to induce cachexia and reduce brain weight (21), here we confirmed significant body weight loss and observed a significant reduction in brain volume with imaging in Pa04C tumor-bearing mice compared to NTB normal mice and non-cachexia inducing Panc1 tumor-bearing mice. These results suggest that a reduction of brain size contributed to the previously identified loss in brain weight (21). While the effects of PDAC on the brain volume have not been previously reported in patients, a recent study based on voxel-based morphometry and functional connectivity (FC) analysis demonstrated that lung cancer patients with bone metastasis exhibited decreased gray matter volume as compared to healthy controls (41). Independent of cachexia, other studies have demonstrated that patients with non-central nervous system (CNS) cancers can exhibit brain abnormalities, including a reduction in gray matter volume (42, 43). For instance, studies with T1-weighted and diffusion tensor MRI imaging have demonstrated that lung cancer patients exhibit reduced gray matter density and white matter density compared to healthy controls (42). Similarly, investigations into breast cancer patients before chemotherapy revealed reduced white matter volume and total brain volume compared to healthy controls (43). These clinical studies suggest that cancers can cause abnormalities in brain volume, even in the absence of cachexia.

The response to the oxygen breathing challenge was clearly compromised in Pa04C tumor-bearing cachectic mice compared to NTB normal mice and Panc1 tumor-bearing non-cachectic mice. Data from previous studies suggests that static optoacoustic biomarkers, such as sO_2_^MSOT^(O_2_) and sO_2_^MSOT^(Air), display minimal correlation with perfusion or hypoxia (39), whereas ΔsO_2_^MSOT^ obtained with OE-MSOT is closely associated with perfusion and hypoxia (38). This inability to of the brain vasculature to increase oxygenation in response to oxygen breathing may have been caused by the reduction of RBCs as anemia is frequently observed in PDAC cachexia (24, 25). Reduced RBCs can result in a limited ability to increase vascular oxygenation in response to oxygen breathing. Chronic inflammation, a characteristic of cancer, disrupts the normal homeostasis of iron distribution and utilization that negatively impacts erythropoiesis in the spleen (44). The spleen is a major reservoir for RBCs (45), and a major storage site for iron. Cachexia-mediated splenic effects may contribute to RBC reduction.

Inflammatory cytokines, including tumor necrosis factor-alpha (TNF-alpha) and interleukins, are often elevated in cachectic individuals (46, 47). We found that the glucocorticoid response element was switched on in the muscle of Pa04C tumor-bearing mice suggesting an elevation of glucocorticoids (18). These cytokines can exert detrimental effects on the vascular system, potentially leading to compromised blood flow and oxygen supply to the brain. In a previous study, a correlation was found between cancer cachexia and neuroinflammatory changes in the brains of Wistar rats with intra-abdominal fibrosarcoma (48). In another study, inflammatory transcripts primarily comprised of chemokines were found to be upregulated in the central nervous system during the growth of PDAC (49). Inflammatory cytokines can cause vascular dysfunction and vascular disease (50). Furthermore, increased brain infiltration of neutrophils has been observed in a mouse model of PDAC (49). The accumulation of infiltrating neutrophils in the brain has been associated with the dysfunction of central nervous system barriers and the stalling of capillary blood flow, resulting in reduced blood perfusion to associated brain regions (51–53).

In our study, no significant correlation was observed between brain volume and brain oxygenation. Brain volume reduction in cachexia was likely multifactorial, involving neuroinflammation and metabolic alterations that occurred over a period of time. Oxygen saturation levels primarily reflected cerebral vascular function and hemodynamics at the time of imaging. While both parameters may be influenced by systemic changes associated with cachexia, their temporal dynamics and underlying mechanisms may be different.

A significant increase in the percent CD31-positive fractional area in the brains of Pa04C tumor-bearing mice compared to NTB normal mice was identified. In contrast, no significant differences in CD31 expression were observed between Panc1 tumor-bearing mice and NTB normal mice, indicating that the increased CD31 expression is likely specific to cachexia-related changes rather than tumor presence alone. Increased expression of CD31, a marker of endothelial cell activity and leukocyte transmigration (54, 55) in the brains of cachectic mice may have been caused by cachexia mediated neuroinflammation (56–59). The contribution of increased CD31 to the hemodynamic changes observed here remains to be determined in future studies.

One limitation of our study is the relatively small sample size. Another limitation is that although we observed weight loss, and previously confirmed activation of the MuRF1 promoter in the Pa04C model, we did not directly measure skeletal muscle mass loss. Incorporating such measurements in future studies to relate to changes in brain hemodynamics would provide a more comprehensive understanding of cachexia.

In conclusion, our data revealed a significant reduction in the brain vasculature response to oxygen breathing in a cachexia inducing PDAC xenograft, identifying a new mechanism that may contribute to cachexia-induced morbidity. Improving brain oxygenation may assist with reducing morbidity.

## Data Availability

The raw data supporting the conclusions of this article will be made available by the corresponding author, without undue reservation.

## References

[1] von Haehling S Anker MS Anker SD. Prevalence and clinical impact of cachexia in chronic illness in europe, USA, and Japan: facts and numbers update 2016. J Cachexia Sarcopenia Muscle. (2016) 7:507–9. doi: 10.1002/jcsm.12167, PMID: 27891294 PMC5114624

[2] Roeland EJ Bohlke K Baracos VE Bruera E Del Fabbro E Dixon S. Management of cancer cachexia: asco guideline. J Clin Oncol Off J Am Soc Clin Oncol. (2020) 38:2438–53. doi: 10.1200/jco.20.00611, PMID: 32432946

[3] Fearon K Strasser F Anker SD Bosaeus I Bruera E Fainsinger RL. Definition and classification of cancer cachexia: an international consensus. Lancet Oncol. (2011) 12:489–95. doi: 10.1016/s1470-2045(10)70218-7, PMID: 21296615

[4] Hendifar AE Chang JI Huang BZ Tuli R Wu BU. Cachexia, and not obesity, prior to pancreatic cancer diagnosis worsens survival and is negated by chemotherapy. J Gastrointestinal Oncol. (2018) 9:17–23. doi: 10.21037/jgo.2017.11.10, PMID: 29564167 PMC5848037

[5] Zhu R Liu Z Jiao R Zhang C Yu Q Han S. Updates on the pathogenesis of advanced lung cancer-induced cachexia. Thorac Cancer. (2019) 10:8–16. doi: 10.1111/1759-7714.12910, PMID: 30461213 PMC6312840

[6] Rich NE Phen S Desai N Mittal S Yopp AC Yang JD. Cachexia is prevalent in patients with hepatocellular carcinoma and associated with worse prognosis. Clin Gastroenterol Hepatol: Off Clin Pract J Am Gastroenterol Assoc. (2022) 20:e1157–e69. doi: 10.1016/j.cgh.2021.09.022, PMID: 34555519 PMC8934317

[7] Callaway CS Mouchantat LM Bitler BG Bonetto A. Mechanisms of ovarian cancer-associated cachexia. Endocrinology. (2023) 165:bqad176. doi: 10.1210/endocr/bqad176, PMID: 37980602 PMC10699881

[8] Kasprzak A. The role of tumor microenvironment cells in colorectal cancer (Crc) cachexia. Int J Mol Sci. (2021) 22(4):1565. doi: 10.3390/ijms22041565, PMID: 33557173 PMC7913937

[9] Muthanandam S Muthu J. Understanding cachexia in head and neck cancer. Asia-Pacific J Oncol Nurs. (2021) 8:527–38. doi: 10.4103/apjon.apjon-2145, PMID: 34527782 PMC8420913

[10] Tan CR Yaffee PM Jamil LH Lo SK Nissen N Pandol SJ. Pancreatic cancer cachexia: A review of mechanisms and therapeutics. Front Physiol. (2014) 5:88. doi: 10.3389/fphys.2014.00088, PMID: 24624094 PMC3939686

[11] Bachmann J Heiligensetzer M Krakowski-Roosen H Büchler MW Friess H Martignoni ME. Cachexia worsens prognosis in patients with resectable pancreatic cancer. J Gastrointestinal Surg: Off J Soc Surg Alimentary Tract. (2008) 12:1193–201. doi: 10.1007/s11605-008-0505-z, PMID: 18347879

[12] Kordes M Larsson L Engstrand L Löhr JM. Pancreatic cancer cachexia: three dimensions of a complex syndrome. Br J Cancer. (2021) 124:1623–36. doi: 10.1038/s41416-021-01301-4, PMID: 33742145 PMC8110983

[13] Danai LV Babic A Rosenthal MH Dennstedt EA Muir A Lien EC. Altered exocrine function can drive adipose wasting in early pancreatic cancer. Nature. (2018) 558:600–4. doi: 10.1038/s41586-018-0235-7, PMID: 29925948 PMC6112987

[14] van Duijneveldt G Griffin MDW Putoczki TL. Emerging roles for the il-6 family of cytokines in pancreatic cancer. Clin Sci (London England: 1979). (2020) 134:2091–115. doi: 10.1042/cs20191211, PMID: 32808663 PMC7434989

[15] Yako YY Kruger D Smith M Brand M. Cytokines as biomarkers of pancreatic ductal adenocarcinoma: A systematic review. PloS One. (2016) 11:e0154016. doi: 10.1371/journal.pone.0154016, PMID: 27170998 PMC4865360

[16] Lira FS Yamashita AS Rosa JC Tavares FL Caperuto E Carnevali LC Jr.. Hypothalamic inflammation is reversed by endurance training in anorectic-cachectic rats. Nutr Metab. (2011) 8:60. doi: 10.1186/1743-7075-8-60, PMID: 21861927 PMC3257200

[17] Plata-Salamán CR Ilyin SE Gayle D. Brain cytokine mrnas in anorectic rats bearing prostate adenocarcinoma tumor cells. Am J Physiol. (1998) 275:R566–73. doi: 10.1152/ajpregu.1998.275.2.R566, PMID: 9688694

[18] Winnard PT Jr. Bharti SK Penet MF Marik R Mironchik Y Wildes F. Detection of pancreatic cancer-induced cachexia using a fluorescent myoblast reporter system and analysis of metabolite abundance. Cancer Res. (2016) 76:1441–50. doi: 10.1158/0008-5472.Can-15-1740, PMID: 26719527 PMC4794402

[19] Bodine SC Latres E Baumhueter S Lai VK Nunez L Clarke BA. Identification of ubiquitin ligases required for skeletal muscle atrophy. Sci (New York NY). (2001) 294:1704–8. doi: 10.1126/science.1065874, PMID: 11679633

[20] Lord SO Dawson PWJ Chunthorng-Orn J Ng J Baehr LM Hughes DC. Uncovering the mechanisms of murf1-induced ubiquitylation and revealing similarities with murf2 and murf3. Biochem Biophysics Rep. (2024) 37:101636. doi: 10.1016/j.bbrep.2023.101636, PMID: 38283190 PMC10818185

[21] Winnard PT Jr. Bharti SK Sharma RK Krishnamachary B Mironchik Y Penet MF. Brain metabolites in cholinergic and glutamatergic pathways are altered by pancreatic cancer cachexia. J Cachexia Sarcopenia Muscle. (2020) 11:1487–500. doi: 10.1002/jcsm.12621, PMID: 33006443 PMC7749557

[22] Yoo HC Park SJ Nam M Kang J Kim K Yeo JH. A variant of slc1a5 is a mitochondrial glutamine transporter for metabolic reprogramming in cancer cells. Cell Metab. (2020) 31:267–83.e12. doi: 10.1016/j.cmet.2019.11.020, PMID: 31866442

[23] Yoo HC Yu YC Sung Y Han JM. Glutamine reliance in cell metabolism. Exp Mol Med. (2020) 52:1496–516. doi: 10.1038/s12276-020-00504-8, PMID: 32943735 PMC8080614

[24] Osmola M Gierej B Mleczko-Sanecka K Jończy A Ciepiela O Kraj L. Anemia, iron deficiency, and iron regulators in pancreatic ductal adenocarcinoma patients: A comprehensive analysis. Curr Oncol (Toronto Ont). (2023) 30:7722–39. doi: 10.3390/curroncol30080560, PMID: 37623041 PMC10453218

[25] Ludwig H Müldür E Endler G Hübl W. Prevalence of iron deficiency across different tumors and its association with poor performance status, disease status and anemia. Ann Oncol Off J Eur Soc Med Oncol. (2013) 24:1886–92. doi: 10.1093/annonc/mdt118, PMID: 23567147 PMC3690908

[26] Ma R Taruttis A Ntziachristos V Razansky D. Multispectral optoacoustic tomography (Msot) scanner for whole-body small animal imaging. Optics Express. (2009) 17:21414–26. doi: 10.1364/oe.17.021414, PMID: 19997381

[27] Ni R Vaas M Ren W Klohs J. Noninvasive detection of acute cerebral hypoxia and subsequent matrix-metalloproteinase activity in a mouse model of cerebral ischemia using multispectral-optoacoustic-tomography. Neurophotonics. (2018) 5:15005. doi: 10.1117/1.NPh.5.1.015005, PMID: 29531962 PMC5829216

[28] Burton NC Patel M Morscher S Driessen WH Claussen J Beziere N. Multispectral opto-acoustic tomography (Msot) of the brain and glioblastoma characterization. NeuroImage. (2013) 65:522–8. doi: 10.1016/j.neuroimage.2012.09.053, PMID: 23026761

[29] Tzoumas S Nunes A Olefir I Stangl S Symvoulidis P Glasl S. Eigenspectra optoacoustic tomography achieves quantitative blood oxygenation imaging deep in tissues. Nat Commun. (2016) 7:12121. doi: 10.1038/ncomms12121, PMID: 27358000 PMC4931322

[30] Olefir I Ghazaryan A Yang H Malekzadeh-Najafabadi J Glasl S Symvoulidis P. Spatial and spectral mapping and decomposition of neural dynamics and organization of the mouse brain with multispectral optoacoustic tomography. Cell Rep. (2019) 26:2833–46.e3. doi: 10.1016/j.celrep.2019.02.020, PMID: 30840901 PMC6403416

[31] Park SJ Ho CJH Arai S Samanta A Olivo M Chang YT. Visualizing alzheimer’s disease mouse brain with multispectral optoacoustic tomography using a fluorescent probe, cdnir7. Sci Rep. (2019) 9:12052. doi: 10.1038/s41598-019-48329-4, PMID: 31427599 PMC6700105

[32] Wang LV Hu S. Photoacoustic tomography: *in vivo* imaging from organelles to organs. Sci (New York NY). (2012) 335:1458–62. doi: 10.1126/science.1216210, PMID: 22442475 PMC3322413

[33] Ramos-Vega M Kjellman P Todorov MI Kylkilahti TM Bäckström BT Ertürk A. Mapping of neuroinflammation-induced hypoxia in the spinal cord using optoacoustic imaging. Acta Neuropathol Commun. (2022) 10:51. doi: 10.1186/s40478-022-01337-4, PMID: 35410629 PMC8996517

[34] Karlas A Kallmayer M Fasoula NA Liapis E Bariotakis M Krönke M. Multispectral optoacoustic tomography of muscle perfusion and oxygenation under arterial and venous occlusion: A human pilot study. J Biophot. (2020) 13:e201960169. doi: 10.1002/jbio.201960169, PMID: 32134550

[35] Merdasa A Bunke J Naumovska M Albinsson J Erlöv T Cinthio M. Photoacoustic imaging of the spatial distribution of oxygen saturation in an ischemia-reperfusion model in humans. Biomed Optics Express. (2021) 12:2484–95. doi: 10.1364/boe.418397, PMID: 33996242 PMC8086473

[36] Liu S Zhang R Han T Pan Y Zhang G Long X. Validation of photoacoustic/ultrasound dual imaging in evaluating blood oxygen saturation. Biomed Optics Express. (2022) 13:5551–70. doi: 10.1364/boe.469747, PMID: 36425613 PMC9664893

[37] Karlas A Fasoula NA Katsouli N Kallmayer M Sieber S Schmidt S. Skeletal muscle optoacoustics reveals patterns of circulatory function and oxygen metabolism during exercise. Photoacoustics. (2023) 30:100468. doi: 10.1016/j.pacs.2023.100468, PMID: 36950518 PMC10025091

[38] Tomaszewski MR Gonzalez IQ O’Connor JP Abeyakoon O Parker GJ Williams KJ. Oxygen enhanced optoacoustic tomography (Oe-ot) reveals vascular dynamics in murine models of prostate cancer. Theranostics. (2017) 7:2900–13. doi: 10.7150/thno.19841, PMID: 28824724 PMC5562224

[39] Tomaszewski MR Gehrung M Joseph J Quiros-Gonzalez I Disselhorst JA Bohndiek SE. Oxygen-enhanced and dynamic contrast-enhanced optoacoustic tomography provide surrogate biomarkers of tumor vascular function, hypoxia, and necrosis. Cancer Res. (2018) 78:5980–91. doi: 10.1158/0008-5472.Can-18-1033, PMID: 30115696

[40] Goggins E Mironchik Y Kakkad S Jacob D Wildes F Bhujwalla ZM. Reprogramming of vegf-mediated extracellular matrix changes through autocrine signaling. Cancer Biol Ther. (2023) 24:2184145. doi: 10.1080/15384047.2023.2184145, PMID: 37389973 PMC10012930

[41] Tang Y Shi Y Xu Z Hu J Zhou X Tan Y. Altered gray matter volume and functional connectivity in lung cancer patients with bone metastasis pain. J Neurosci Res. (2024) 102:1–12. doi: 10.1002/jnr.25256, PMID: 38284835

[42] Simó M Root JC Vaquero L Ripollés P Jové J Ahles T. Cognitive and brain structural changes in a lung cancer population. J Thorac Oncol Off Publ Int Assoc Study Lung Cancer. (2015) 10:38–45. doi: 10.1097/jto.0000000000000345, PMID: 25325778 PMC5657249

[43] Scherling C Collins B Mackenzie J Lepage C Bielajew C Smith A. Structural brain differences in breast cancer patients compared to matched controls prior to chemotherapy. J Int Biol. (2012) 4:3–25. doi: 10.5539/ijb.v4n2p3

[44] Chang J Debreli Coskun M Kim J. Inflammation alters iron distribution in bone and spleen in mice. Metallomics: Integrated Biometal Sci. (2023) 15(10):mfad055. doi: 10.1093/mtomcs/mfad055, PMID: 37738439 PMC10563149

[45] Lindblom H Pernett F Schagatay E Holmström P. Effect of exercise intensity and apnea on splenic contraction and hemoglobin increase in well-trained cross-country skiers. Eur J Appl Physiol. (2024) 124:2057–67. doi: 10.1007/s00421-024-05428-z, PMID: 38393417 PMC11199288

[46] Riccardi D das Neves RX de Matos-Neto EM Camargo RG Lima J Radloff K. Plasma lipid profile and systemic inflammation in patients with cancer cachexia. Front Nutr. (2020) 7:4. doi: 10.3389/fnut.2020.00004, PMID: 32083092 PMC7005065

[47] Webster JM Kempen L Hardy RS Langen RCJ. Inflammation and skeletal muscle wasting during cachexia. Front Physiol. (2020) 11:597675. doi: 10.3389/fphys.2020.597675, PMID: 33329046 PMC7710765

[48] Cernackova A Tillinger A Bizik J Mravec B Horvathova L. Dynamics of cachexia-associated inflammatory changes in the brain accompanying intra-abdominal fibrosarcoma growth in wistar rats. J Neuroimmunol. (2023) 376:578033. doi: 10.1016/j.jneuroim.2023.578033, PMID: 36738563

[49] Burfeind KG Zhu X Norgard MA Levasseur PR Huisman C Buenafe AC. Circulating myeloid cells invade the central nervous system to mediate cachexia during pancreatic cancer. eLife. (2020) 9:e54095. doi: 10.7554/eLife.54095, PMID: 32391790 PMC7253193

[50] Singh SK Singh R. Cytokines and chemokines in cancer cachexia and its long-term impact on covid-19. Cells. (2022) 11:579. doi: 10.3390/cells11030579, PMID: 35159388 PMC8834385

[51] Aries ML Hensley-McBain T. Neutrophils as a potential therapeutic target in alzheimer’s disease. Front Immunol. (2023) 14:1123149. doi: 10.3389/fimmu.2023.1123149, PMID: 36936930 PMC10020508

[52] Santos-Lima B Pietronigro EC Terrabuio E Zenaro E Constantin G. The role of neutrophils in the dysfunction of central nervous system barriers. Front Aging Neurosci. (2022) 14:965169. doi: 10.3389/fnagi.2022.965169, PMID: 36034148 PMC9404376

[53] El Amki M Glück C Binder N Middleham W Wyss MT Weiss T. Neutrophils obstructing brain capillaries are a major cause of no-reflow in ischemic stroke. Cell Rep. (2020) 33:108260. doi: 10.1016/j.celrep.2020.108260, PMID: 33053341

[54] Almutairi MM Gong C Xu YG Chang Y Shi H. Factors controlling permeability of the blood-brain barrier. Cell Mol Life Sci. (2016) 73:57–77. doi: 10.1007/s00018-015-2050-8, PMID: 26403789 PMC11108286

[55] Albelda SM Smith CW Ward PA. Adhesion molecules and inflammatory injury. FASEB J. (1994) 8:504–12. doi: 10.1096/fasebj.8.8.8181668 8181668

[56] Kalinowska A Losy J. Pecam-1, a key player in neuroinflammation. Eur J Neurol. (2006) 13:1284–90. doi: 10.1111/j.1468-1331.2006.01640.x, PMID: 17116209

[57] Preuss SF Grieshober D Augustin HG. Systemic reprogramming of endothelial cell signaling in metastasis and cachexia. Physiol (Bethesda Md). (2023) 38. doi: 10.1152/physiol.00001.2023, PMID: 37222464 PMC10281790

[58] Wu F Zhao Y Jiao T Shi D Zhu X Zhang M. Cxcr2 is essential for cerebral endothelial activation and leukocyte recruitment during neuroinflammation. J Neuroinflamm. (2015) 12:98. doi: 10.1186/s12974-015-0316-6, PMID: 25990934 PMC4438521

[59] Takata F Nakagawa S Matsumoto J Dohgu S. Blood-brain barrier dysfunction amplifies the development of neuroinflammation: understanding of cellular events in brain microvascular endothelial cells for prevention and treatment of bbb dysfunction. Front Cell Neurosci. (2021) 15:661838. doi: 10.3389/fncel.2021.661838, PMID: 34588955 PMC8475767

