# Supplementary material for: Optoacoustic imaging of the brain in a cachexia-inducing pancreatic cancer xenograft
Source: Front Oncol. 2025 Oct 13;15:1580640. doi: 10.3389/fonc.2025.1580640 (PMC12554560; doi:10.3389/fonc.2025.1580640)
Supplement: Supplementary file 1 [file DataSheet1.docx]

Supplementary Material

**Optoacoustic imaging of the brain in a cachexia-inducing pancreatic cancer xenograft**

Saleem Yousf^1^, Marie-France Penet^1,2^, Andrew Brannen^3^, Paul Winnard^1^, Yelena Mironchik^1^, Balaji Krishnamachary^1,2^, Zaver M Bhujwalla^1, 2, 4@^

^1^Division of Cancer Imaging Research, The Russell H. Morgan Department of Radiology and Radiological Science, The Johns Hopkins University School of Medicine, Baltimore, MD, USA

^2^Sidney Kimmel Comprehensive Cancer Center, The Johns Hopkins University School of Medicine, Baltimore, MD, USA

^3^iThera Medical, Munich, Germany

^4^Department of Radiation Oncology and Molecular Radiation Sciences, The Johns Hopkins University School of Medicine, Baltimore, MD, USA

@Correspondence:

Zaver M. Bhujwalla, PhD

Department of Radiology

The Johns Hopkins University School of Medicine

720 Rutland Avenue, Rm 208C Traylor Building

Baltimore, MD 21205, USA

Email: zbhujwa1@jhmi.edu

**Running title**: Cachexia alters brain hemodynamics


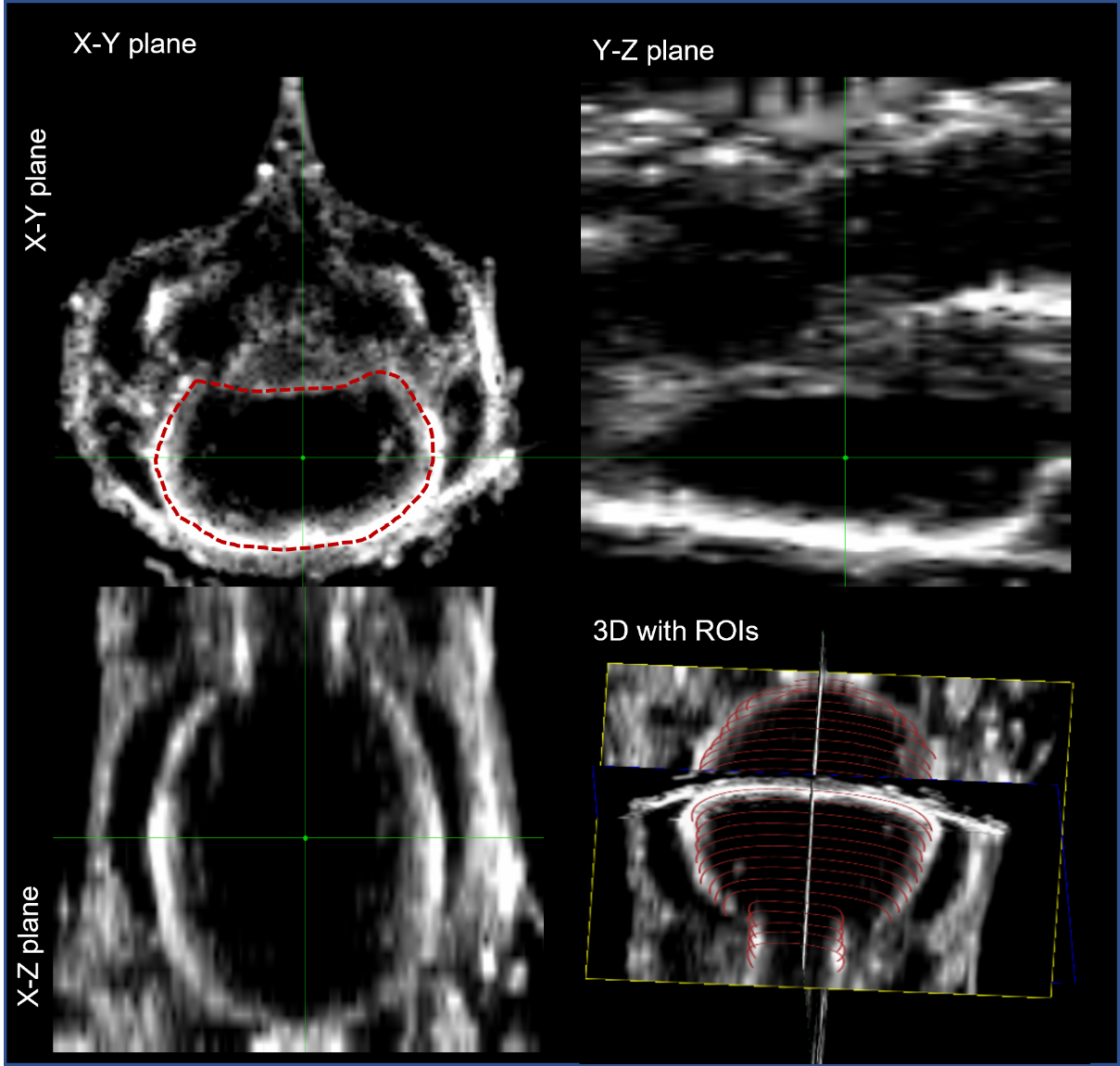


**Figure S1:** Representative example of delineation of the 3D region of interest (ROI) for brain volume quantification. Orthogonal planes in the X-Y, X-Z, and Y-Z orientations were used to define the 3D ROI, encompassing the entire brain. ROIs delineated using ultrasound images, are shown by the red dashed lines in the X-Y plane and visualized in 3D (bottom right). The figure demonstrates the application of these ROIs in different planes, allowing for precise 3D brain volume quantification and visualization.

| **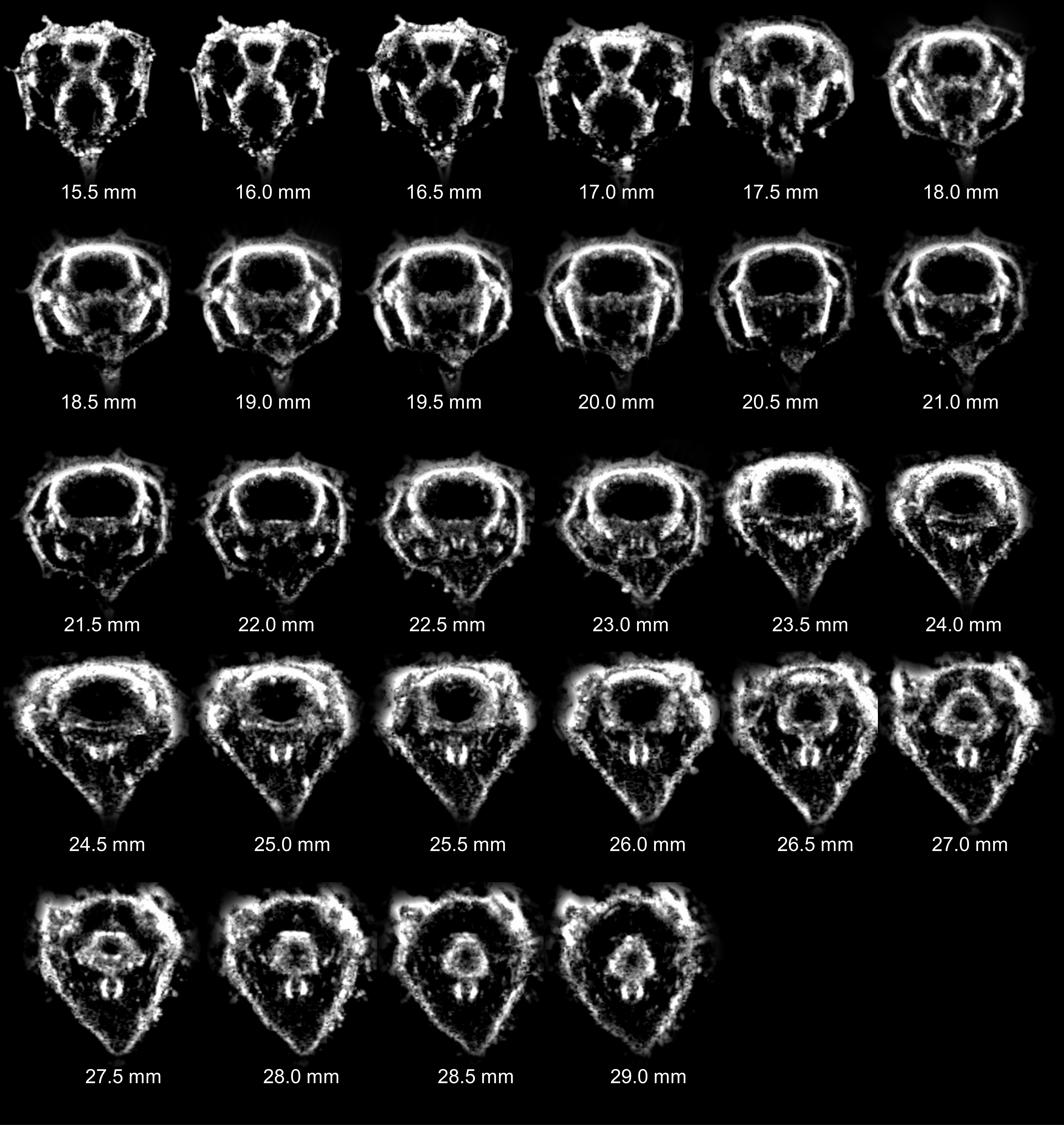** |
| --- |

**Figure S2:** Coronal slices of the representative mouse brain presented in Figure 2B obtained in B-mode ultrasound using the MSOT scanner. Images were acquired at 0.5 mm step intervals.


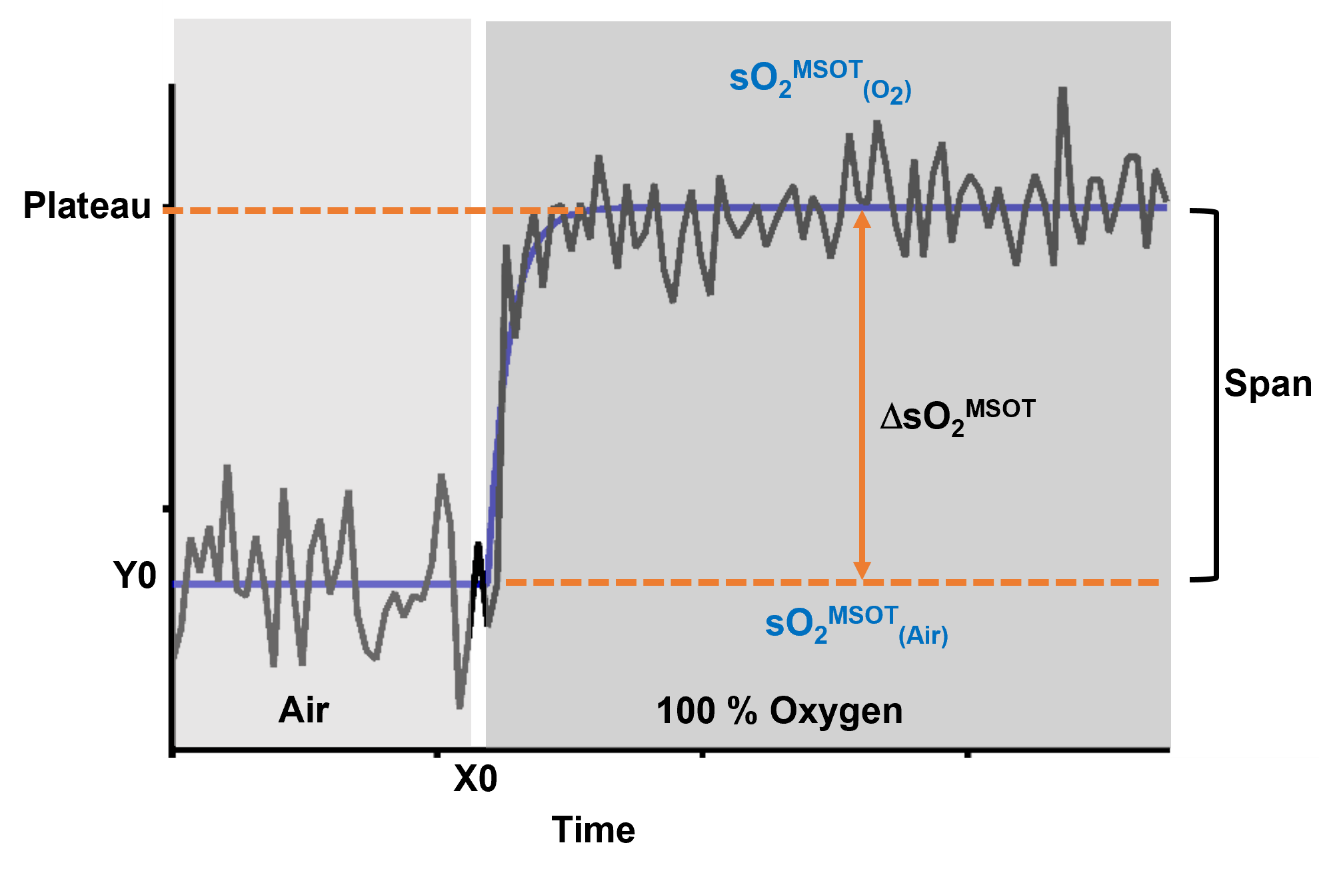


**Figure S3: Curve fitting of OE-MSOT signal.** Data were fitted to a plateau followed by a one-phase association equation to extract OE-MSOT metrics. These metrics include baseline sO_2_^MSOT^ values before [sO_2_^MSOT^(Air)] and after the oxygen challenge [sO_2_^MSOT^(O_2_)], as well as the amplitude of signal change (ΔsO_2_^MSOT^). The x-axis represents time, and the y-axis represents mean oxygen saturation (Mean sO_2_^MSOT^).

.

.


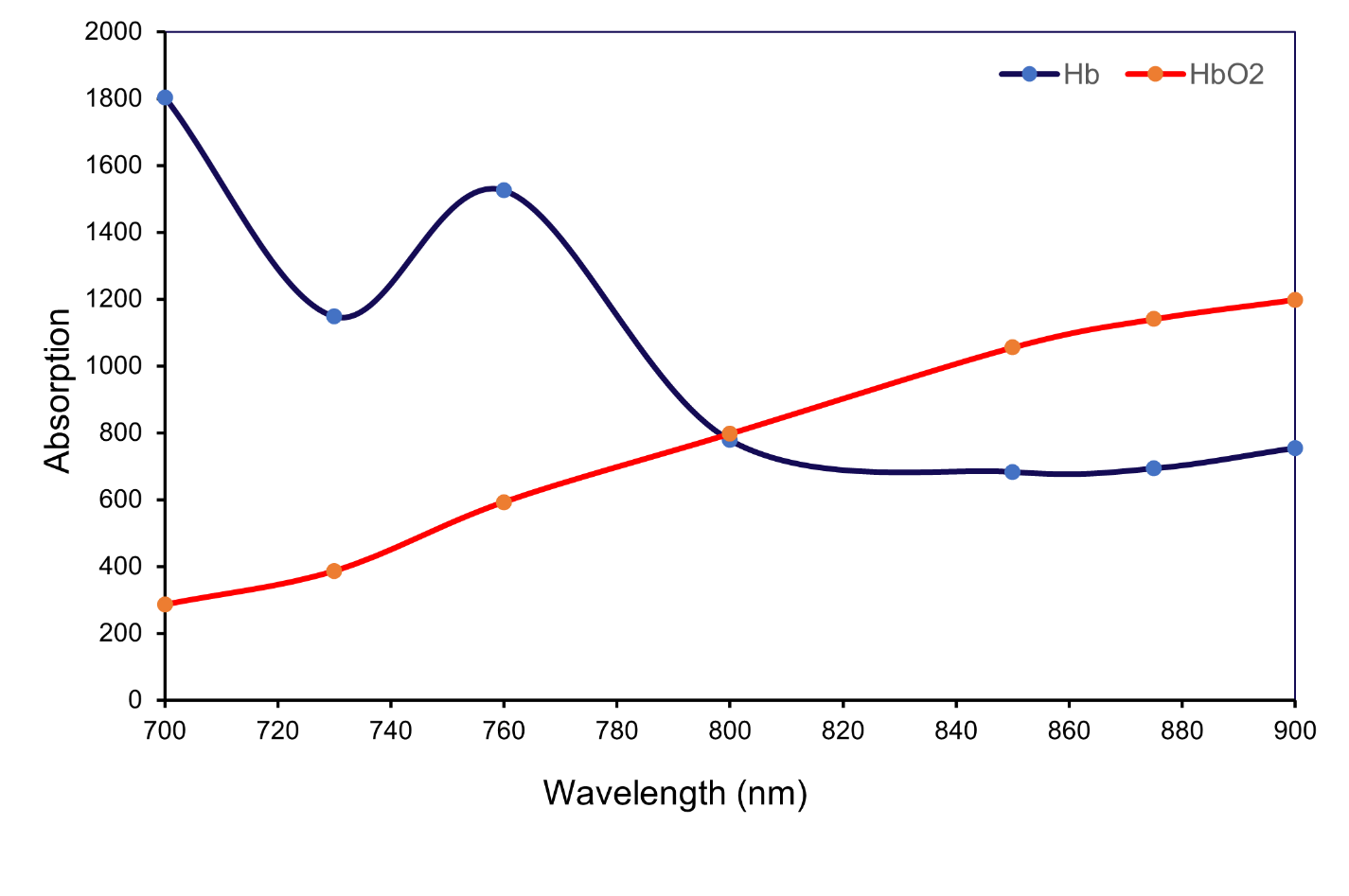


**Figure S4:** The absorption spectrum of Hb and HbO_2_.


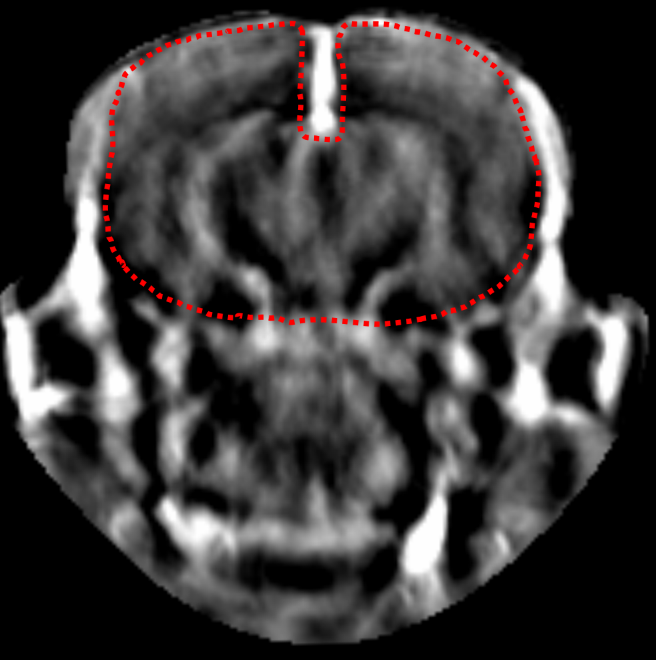


**Figure S5:** Representative region of interest (ROI) delineated for oxygenation quantification.
